# Cerebral inflammatory amyloid angiopathy: response to treatment

**DOI:** 10.1590/1980-5764-DN-2024-0263

**Published:** 2025-08-18

**Authors:** João Roberto Ribeiro Pimenta, Júlian Letícia Freitas, Maria Sheila Guimarães Rocha, Sonia Maria Dozzi Brucki

**Affiliations:** 1 Hospital Santa Marcelina, Departamento de Neurologia, São Paulo SP, Brazil.

**Keywords:** Cerebral Amyloid Angiopathy, Amyloid Beta-Peptides, Immunosuppression Therapy, Magnetic Resonance Imaging, Angiopatia Amiloide Cerebral, Peptídeos beta-Amiloides, Terapia de Imunossupressão, Imageamento por Ressonância Magnética

## Abstract

Cerebral Amyloid Angiopathy (CAA) is a small vessel disease associated with β-amyloid (Aβ) deposition in cortical and leptomeningeal vessels. Traditionally diagnosed through invasive methods, it can now be identified via advanced imaging modalities, enhancing non-invasive diagnostic accuracy. A subset of patients exhibits an inflammatory presentation, termed Inflammatory Cerebral Amyloid Angiopathy (CAA-RI), characterized by cognitive decline, behavioral changes, and neurological deficits. This study highlighted two cases of CAA-RI with subacute onset, detailed clinical progression, and distinct MRI findings consistent with revised diagnostic criteria, enabling early suspicion. Both cases showed significant improvement with immunosuppressive therapy, reinforcing the potentially reversible nature of CAA-RI and the importance of early recognition. This article underscores the relevance of advanced imaging in the differential diagnosis of CAA and the potential for improved patient outcomes with timely treatment.

## RESUMO

Angiopatia Amiloide Cerebral (AAC) é uma doença de pequenos vasos associada ao depósito de β-amiloide (Aβ) nos vasos corticais e leptomeníngeos. Tradicionalmente diagnosticada por métodos invasivos, atualmente pode ser identificada por meio de técnicas avançadas de imagem, o que aumenta a precisão diagnóstica de forma não invasiva. Um subgrupo de pacientes apresenta uma forma inflamatória, denominada Angiopatia Amiloide Cerebral Inflamatória (AAC-RI), caracterizada por declínio cognitivo, alterações comportamentais e déficits neurológicos. Este estudo destacou dois casos de AAC-RI com início subagudo, detalhou a progressão clínica e apresentou achados distintos de ressonância magnética compatíveis com os critérios diagnósticos revisados, permitindo o diagnóstico precoce. Ambos os casos apresentaram melhora significativa com terapia imunossupressora, reforçando o potencial de reversibilidade da AAC-RI e a importância do reconhecimento precoce. Este artigo enfatiza a relevância das técnicas de imagem avançadas no diagnóstico diferencial da AAC e o potencial de melhores desfechos clínicos com tratamento oportuno.

## INTRODUCTION

 Cerebral amyloid angiopathy (CAA) is an age-related small vessel disease affecting cortical and leptomeningeal vessels, pathologically characterized by the progressive deposition of β-amyloid (Aβ) in the vascular wall^1,2^. Aβ deposition is centrally associated with cerebral microbleeds (CMB) and lobar intracerebral hemorrhages (ICH), attributed to vascular fragility due to protein aggregation. Non-hemorrhagic manifestations such as cognitive decline, dementia, cortical atrophy, leukoencephalopathy, and transient focal neurological deficits have recently gained recognition as part of the clinical spectrum^1,3^. Typically, the diagnosis of CAA relies on demonstrating Aβ birefringence with Congo red staining viewed under polarized light microscopy in a brain biopsy^1,4,5^. However, advances in imaging have improved diagnostic accuracy, enabling non-invasive identification of CAA through clinical-radiological features such as CMB, cortical atrophy, and superficial siderosis^5^. To support diagnosis without invasive procedures, Charidimou et al.^6^ reviewed the previously established Boston criteria, incorporating magnetic resonance imaging (MRI) findings to improve accuracy. The study included patients with hemorrhagic syndromes, such as ICH, as well as non-hemorrhagic syndromes, specifically dementia/cognitive impairment and transient focal neurological episodes related to CAA. The presence of cortical superficial siderosis (cSS), convexity subarachnoid hemorrhage (cSAH), or white matter changes associated with CAA, when combined with a hemorrhagic marker (ICH, CMB, cSS, cSAH) in patients over 50 years of age within this clinical spectrum, demonstrated greater accuracy compared to the previous criteria. Moreover, the revised criteria maintained high sensitivity without increasing false-positive rates relative to the reference standard of invasive diagnosis via biopsy, histopathological analysis, or autopsy^6^. 

 Additionally, a rarer presentation marked by subacute encephalopathy, behavioral changes, headache, and seizures appears to be associated with inflammatory vasculopathy, differing from the more commonly observed manifestations. The first report of vascular inflammation related to Aβ was described in 1974 by Reid and Maloney, initially in patients with Alzheimer disease (AD)^7^. Since then, several studies have independently demonstrated an association between this clinical spectrum and pathological findings unrelated to AD. The alterations observed include confluent hyperintensities on MRI T2 and T2 FLAIR sequences, which may be symmetric or asymmetric and occasionally involve contrast enhancement, suggesting edema as part of the pathophysiology^4,8^. This presentation reflects an inflammatory vascular process against Aβ proteins in leptomeningeal and cortical vessels. This process may manifest as non-destructive perivascular inflammation (CAA-related inflammation – CAA-RI) and transmural or intramural inflammation (Aβ-related angiitis – ABRA)^4,9^. Recent findings indicate a high incidence of the apolipoprotein E ε4/ε4 genotype (apoE) among patients with CAA-RI. Epidemiologically, individuals with CAA-RI tend to be younger than those presenting with CAA-RI^8,9^. Understanding the inflammatory form of CAA is crucial, as immunosuppressive treatments have shown varying degrees of clinical recovery, establishing it as a potentially treatable form of dementia requiring high clinical suspicion for rapid diagnosis and treatment initiation^1,8^. Improving recognition of CAA-RI facilitates diagnosis without the need for brain biopsy, which is essential for early intervention. 

 Two cases with inflammatory manifestations of CAA and their response to the selected treatment are presented below. 

## CASE REPORT 1

A 79-year-old right-handed retired male with a history of systemic arterial hypertension and ongoing follow-up with the proctology team for advanced-stage rectal adenocarcinoma (T4N2aM0) presented to our service due to rectal bleeding that had evolved over two weeks. The surgical team noted that the patient was dependent on instrumental activities of daily living (IADLs) and partially on basic activities of daily living (BADLs), requiring a family caregiver. Thus, a neurology consultation was requested. Approximately one year prior, the patient began experiencing difficulty managing payments, checking change, and shopping for groceries, exhibiting some degree of disorientation. Around six months before hospitalization, significant inattentiveness and worsening mental confusion were noted. The patient also displayed impaired ability to perform simple tasks, such as heating food, and exhibited unusual behavior, including placing a plate with cooked beans and uncooked rice directly on the stove flame. Between three to four months prior to hospitalization, the patient ceased recognizing neighbors and stopped regular bathing, only doing so when explicitly instructed, with a marked decline in personal hygiene and self-care. The condition progressively worsened, and by the time of hospitalization, the patient required total assistance with all BADLs.

**Figure 1:**
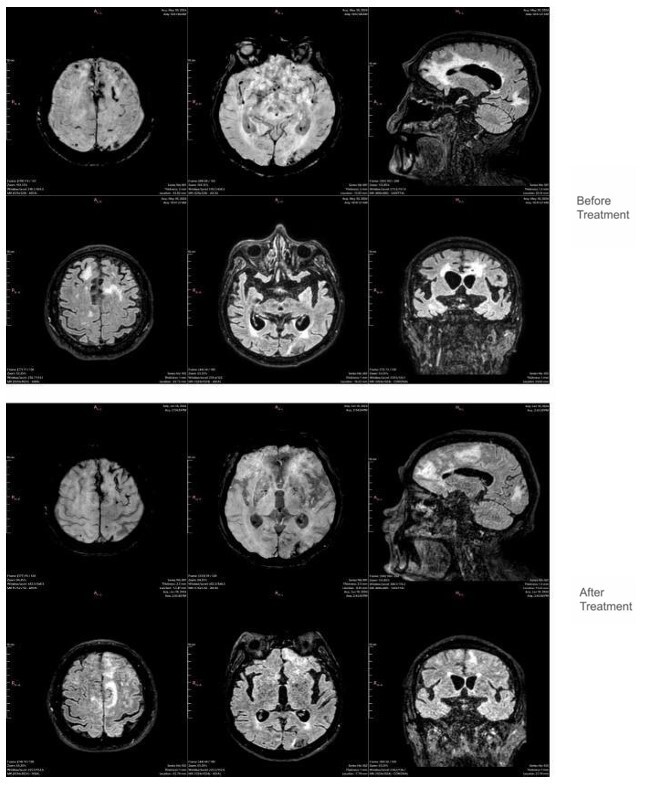
Images related to Case 1 include susceptibility-weighted imaging sequences (the first two figures in the top row) and their corresponding FLAIR sequences (the two figures immediately below). These demonstrate that the hyperintense signals in the FLAIR sequence correspond to areas with the highest concentration of hypointense signals on the susceptibility-weighted imaging sequence, which, in this context, are suggestive of hemorrhage. The rightmost image in the top row shows a sagittal FLAIR sequence, while the rightmost image in the second row displays a coronal FLAIR sequence, providing a better understanding of the extent of inflammation. Post-treatment images, shown in the two bottom rows, represent the same anatomical locations as those in the top rows.

 During the neurological examination, the patient spoke only a few words, was unable to name or repeat objects, partially understood commands, was minimally cooperative, and could not complete formal cognitive tests. There was no dysarthria or cranial nerve abnormality. Although cooperation for strength assessment was limited, no apparent pyramidal deficit or release signs were observed. Cranial computed tomography (CT) revealed accentuated sulci and fissures without regional predominance, a hypoattenuated subcortical area in the left occipital region, and periventricular white matter hypodensities. Cervical and cranial CT angiography performed for vascular assessment showed no hemodynamically significant stenosis or vascular malformation. MRI findings were compatible with inflammatory amyloid angiopathy (Figure 1). Initial laboratory evaluations were within reference ranges, including renal function, electrolytes, complete blood count, liver enzymes, thyroid hormones, and urinalysis. Serologic testing for hepatitis C, HIV, and syphilis were non-reactive. Evaluation for hepatitis B indicated past exposure and resolved infection (non-reactive HBsAg, reactive anti-HBs, and anti-HBc). Vitamin B12 and folic acid levels were within normal limits. Cerebrospinal fluid (CSF) analysis revealed elevated protein levels (140.2 mg/dL), 517.3 red blood cells/mL, 0.3 white blood cells/mL, and a glucose concentration of 58 mg/dL (serum glucose of 86 mg/dL). Cultures, cryptococcal antigen testing, and oncologic cytology were all negative. Electroencephalography (EEG) performed during the initial evaluation demonstrated disorganized baseline activity, poor anterior-posterior differentiation, an irregular excess of slow theta and delta waves, and occasional epileptiform paroxysms with sharp waves projected in the left frontocentral region. The diagnostic workup was conducted approximately 20 days after hospital admission and prior to treatment initiation. The patient was treated with a five-day course of 1g intravenous methylprednisolone, followed by oral prednisone at 1 mg/kg as maintenance, and scheduled to receive monthly methylprednisolone pulses for three months, without adverse events. Progressive clinical improvement was observed following corticosteroid therapy. After diagnosis and initial improvement, the patient redirected his focus toward treatment of advanced rectal neoplasia, which limited follow-up and serial cognitive assessments. Unfortunately, the patient died due to complications related to cancer. 

## CASE REPORT 2

 A 78-year-old right-handed female with a medical history of systemic arterial hypertension, dyslipidemia, diabetes mellitus, and osteoporosis presented to our hospital with acute worsening, beginning five days prior to admission, of a preexisting cognitive impairment that had primarily affected memory over the preceding 60 days. Upon admission, the patient exhibited progressive memory complaints, mental confusion, and visual disturbances. The family reported no history or symptoms suggestive of infection or constitutional symptoms. Prior to the onset of cognitive decline, the patient was independent in both BADLs and IADLs. 

 On initial examination, blood pressure was within normal limits. Neurological evaluation revealed pyramidal release signs in all four limbs, right homonymous hemianopia that progressed to bilateral amaurosis over a few days, and significant cognitive impairment with marked attentional deficits (Table 1). 

**Table 1:** Progression of cognitive test results for Case 2. The patient is illiterate with no formal schooling.

	Patient 2		
	Before treatment	After treatment	After 4 months
MMSE	8/30	17/30	21/30
BCSB-FMT	-	-	10/7/1/0/0/0/4 (-3)*
VF (animals)	2	3	6
Clock drawing	0	1	1

Abbreviations: MMSE, Mini-Mental State Examniation; BCSB-FMT, The Figure Memory Test; VF, Semantic Verbal Fluency.

Notes: *perception/naming/incidental memory/ immediate recall/ learning/delayed recall/recognition.

**Figure 2:**
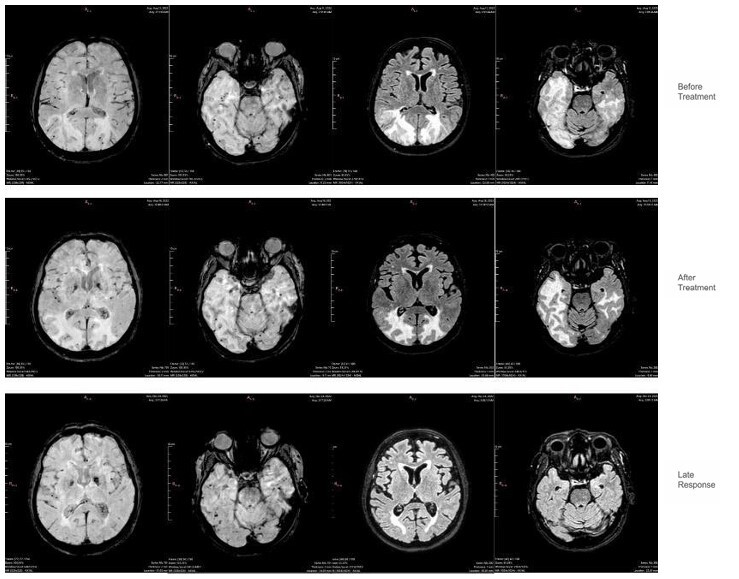
Images related to Case 2 show, from left to right, two susceptibility-weighted imaging- MRI slices and their corresponding FLAIR-weighted slices, highlighting that the hyperintense signal in the latter corresponds to areas with the highest concentration of hypointense signals on the susceptibility-weighted imaging sequence, which in this case are suggestive of hemorrhage. The middle and lower images demonstrate post-treatment evolution and a delayed response.

 Screening tests for secondary causes of cognitive impairment were within normal ranges. CSF analysis revealed elevated protein (197 mg/dL) with normal cell counts and glucose levels. Brain MRI findings are shown in Figure 2. The patient was treated with 1 g of intravenous methylprednisolone for five days, followed by a gradual corticosteroid taper and initiation of azathioprine as a steroid-sparing agent. No adverse events were observed. The patient demonstrated significant neurological improvement, including complete resolution of visual deficits, improvement temporal and spatial orientation, and recognition of family members. Cognitive outcomes are summarized in Table 1. Subsequent CSF analysis, performed four months after the initial test, showed normalization of protein levels (39 mg/dL). 

## DISCUSSION

**Figure 3:**
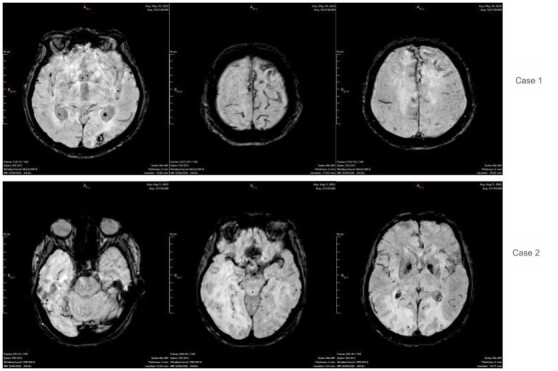
The images show susceptibility-weighted imaging sequences obtained at diagnosis, with the top three corresponding to Case 1 and the bottom three to Case 2. The hypointense signals observed in this context are suggestive of hemorrhagic sites.

 We present two cases of rapidly progressive cognitive and functional decline, initially characterized by attentional deficits, although other domains such as language, executive functions, and memory may also have been affected. Both cases initially underwent cranial CT scans, which revealed indirect signs of nonspecific subcortical edema. Subsequent cranial MRI demonstrated findings consistent with CAA-RI, particularly confluent subcortical T2 hyperintensity and indirect signs of hemorrhage, such as CMB and superficial siderosis (Figure 3). Specific imaging characteristics may have sufficiently high diagnostic accuracy to prevent the need for biopsy confirmation. The most significant imaging feature associated with CAA-RI is a confluent, subcortical, and asymmetric hyperintense signal on T2-weighted MRI sequences^1,4,9,10^. Multiple lobar CMBs located in cortical and subcortical regions that coincide with T2 hyperintensity add high specificity to the diagnosis of CAA-RI^10^. Additionally, superficial siderosis is recognized as an indirect sign of cortical hemorrhage and is therefore included in diagnostic criteria. Furthermore, susceptibility-weighted imaging (SWI) has demonstrated greater sensitivity in identifying microbleeds compared to traditional T2*-gradient echo (T2-GE) and should be routinely employed in the evaluation of suspected CAA-RI cases^5^. Compared with non-inflammatory CAA, the typical occipital predominance seen in the latter is generally absent in CAA-RI. Gadolinium-based contrast enhancement may or may not be present; however, lobar or leptomeningeal enhancement is more frequently observed in CAA-RI than in non-inflammatory forms. Given the involvement of small-caliber arteries, magnetic resonance angiography (MRA) typically does not show significant abnormalities^1,4,9^. By evaluating imaging findings in conjunction with a compatible clinical presentation, it was possible to suspect that both cases fulfilled the diagnostic criteria for CAA-RI without requiring invasive confirmation. 

 Several authors have proposed diagnostic criteria for probable CAA-RI without biopsy, aiming to expedite treatment and reduce the morbidity associated with invasive procedures. Chung et al.^11^ suggested that characteristic clinical symptoms, together with imaging findings indicative of inflammation and classical features of cerebral amyloid angiopathy (*e.g*., CMB), are sufficient to consider a diagnosis of probable CAARI. Auriel et al. revised these criteria, demonstrating a sensitivity of 82% and specificity of 97% for probable CAA-RI^4,10-12^ (Table 2). The criteria include four shared elements (age, clinical symptoms, hemorrhagic signs, and exclusion criteria) between the "possible CAA-RI" and "probable CAA-RI" categories. The distinguishing factor is the pattern of white matter hyperintensity: in probable CAA-RI, it extends immediately into the subcortical space with an asymmetrical distribution, whereas in possible CAA-RI, the extension is present but not necessarily asymmetrical. A recent retrospective study by Grangeon et al.^13^, which analyzed data from a retrospective cohort, reported a lower sensitivity for probable CAA-RI (71%, 95%CI 44–90%) compared to prior studies. This finding suggests that relying on asymmetry in white matter edema may result in missed diagnoses and potentially reduced detection rates when classical diagnostic criteria are applied^14-16^. Both patients 1 and 2 met the revised criteria, and the diagnosis of probable CAA-RI was pivotal for early treatment initiation. Additionally, as observed in both cases, the clinical response to immunosuppressive therapy serves as an essential supportive criterion. 

**Table 2:** Diagnostic Criteria for Inflammatory Cerebral Amyloid Angiopathy.

Essential	Age>40 years
	One or more clinical features: headache, altered level of consciousness, behavioral changes, focal neurological signs, or seizures. The clinical presentation cannot be directly explained by parenchymal hemorrhage (ICH)
	One or more cortico-subcortical hemorrhagic lesions: macrohemorrhages (ICH), microhemorrhages (CMB), or superficial siderosis
	Absence of other causes such as neoplasm or infection
Specific	Probable: MRI demonstrating unilateral or multifocal white matter lesions with an asymmetrical distribution extending to the subcortical white matter; asymmetry cannot be explained by the presence of ICH
	Possible: MRI demonstrating white matter lesions extending immediately to the subcortical white matter

Abbreviations: ICH, intracerebral hemorrhages; CMB, cerebral microbleeds; MRI, magnetic resonance imaging.

Source: Auriel et al.^10^.

 Whether using high-dose corticosteroids alone or in combination with other immunosuppressive agents, the therapeutic response in CCA-RI has proven superior to non-treatment, regardless of the clinical presentation. This contrasts with non-inflammatory CAA, which generally shows no therapeutic response^4,11,13^. A systematic review of CAA-RI revealed that, irrespective of presentation, 55% of patients remained asymptomatic or had only mild disability following immunosuppressive therapy alone or in combination, over a 24-month follow-up^14^. Despite this, the disease typically results in severe functional outcome. Another systematic review reported that up to 60% of patients either died or experienced severe functional impairment after immunotherapy^13^. There remains no clear consensus in the literature regarding the use of corticosteroids alone versus combined immunosuppressive therapy, nor regarding the optimal duration of treatment. Studies have not demonstrated significant differences in outcomes between monotherapy and combination therapy^4,11,13^. 

 In conclusion, the presented cases and accompanying review of diagnostic and therapeutic strategies underscore that, although CAA-RI is rare, it constitutes a significant cause of acute or subacute loss of higher cortical functions. The prognosis is favorable when suspicion arises early and treatment is initiated promptly. Cases presenting with specific clinical features such as headache, altered level of consciousness, behavioral changes, focal neurological signs, or seizures should be evaluated for possible CAA-RI. In this context, contrast-enhanced brain MRI provides excellent accuracy for probable or possible CAA-RI, thereby reducing the need for invasive diagnostic procedures, which are now mainly reserved for cases with diagnostic uncertainty, particularly when differentiating imaging findings from neoplasms. 

## Data Availability

The data that support the findings of this study are available from the corresponding author upon reasonable request.
